# Molecular Breeding to Create Optimized Crops: From Genetic Manipulation to Potential Applications in Plant Factories

**DOI:** 10.3389/fpls.2016.00539

**Published:** 2016-04-25

**Authors:** Kyoko Hiwasa-Tanase, Hiroshi Ezura

**Affiliations:** Graduate School of Life and Environmental Sciences, University of TsukubaTsukuba, Japan

**Keywords:** plant factory, molecular breeding, agricultural crop trait, additional value, molecular farming, edible medicine

## Abstract

Crop cultivation in controlled environment plant factories offers great potential to stabilize the yield and quality of agricultural products. However, many crops are currently unsuited to these environments, particularly closed cultivation systems, due to space limitations, low light intensity, high implementation costs, and high energy requirements. A major barrier to closed system cultivation is the high running cost, which necessitates the use of high-margin crops for economic viability. High-value crops include those with enhanced nutritional value or containing additional functional components for pharmaceutical production or with the aim of providing health benefits. In addition, it is important to develop cultivars equipped with growth parameters that are suitable for closed cultivation. Small plant size is of particular importance due to the limited cultivation space. Other advantageous traits are short production cycle, the ability to grow under low light, and high nutriculture availability. Cost-effectiveness is improved from the use of cultivars that are specifically optimized for closed system cultivation. This review describes the features of closed cultivation systems and the potential application of molecular breeding to create crops that are optimized for cost-effectiveness and productivity in closed cultivation systems.

## Introduction

Plant factories consist of artificially controlled environments for the cultivation of plants within buildings. Agricultural commodities can be efficiently and continuously produced in plant factories regardless of season or external weather conditions. Optimal environmental conditions can be maintained by control of factors such as light intensity, light duration, CO_2_ concentration, and nutrient levels. Plant factories are divided into the following three production types according to their light sources: (1) solar light, (2) combined solar and artificial light, and (3) perfection-artificial-light (Goto, 2011). Solar and combined-light factories are suitable for the cultivation of crops that need intense light. However, it is difficult to use multistage racking in these systems, and the management of pests and diseases is also challenging. Perfection-artificial-light systems, also known as closed cultivation systems, facilitate the maintenance of pest- and disease-free conditions and can therefore be used to produce pesticide-free cultivars. The use of closed cultivation systems to produce genetically modified crops helps prevent the spread of transgenic plants and pollen to the external environment. Closed cultivation systems can also be used to produce cultivars through the use of multistage-cultivation-racks, which make efficient use of limited space, thereby contributing to increased cultivation number and yields per unit area (Kozai, 2013). However, due to the limited light intensity and limited space, such systems are not suitable for every crop. A further disadvantage is the high operating costs resulting from high energy requirements for artificial lighting and air-conditioning (Kozai, 2013).

Production cost is a major factor for plant factories. To be profitable, agricultural cultivation in plant factories is mostly limited to products such as leafy vegetables and flower and vegetable seedlings. Advantages of leafy vegetables, such as lettuce and arugula, include the large proportion of the plant that is edible, year-round demand, high productivity over a short life cycle, and short plant height. Additionally, the energy costs are relatively low because leafy vegetables can grow under low light intensities. High light intensities (photon flux density) can be achieved using high-pressure sodium lamps and metal halide lamps (Goto, 2011). If energy costs were not of concern, it would be possible to dramatically increase the number of crop species that could be cultivated in plant factories by using high-intensity lamps alongside air-conditioning.

In this review, molecular breeding of crops to enhance their suitability for cost-effectiveness in closed cultivation systems is discussed. Preferable crop traits for closed cultivation systems are also suggested in terms of the potential value.

## Increasing the Added Value of Agricultural Products

**FIGURE 1:**
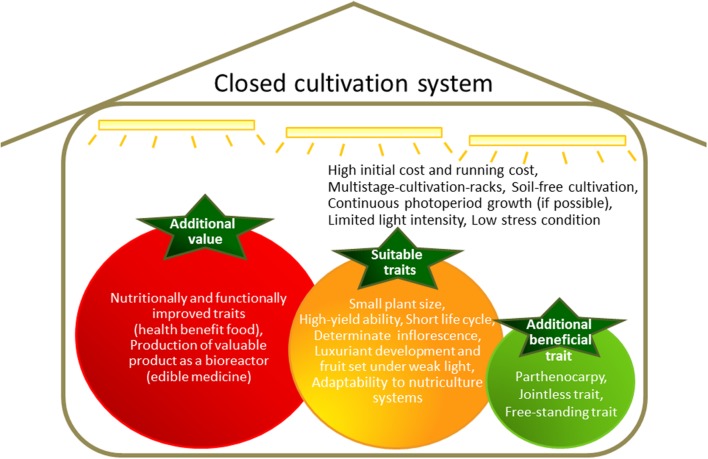
**Features of a closed cultivation system and ideal traits for cultivation in such a system**.

Two major considerations of closed cultivation systems are the initial costs and the running costs, with high energy costs of particular concern (**Figure 1**). Profitability can be increased by producing crops with enhanced value.

### Agricultural Crops with Health Benefits

Two of the main approaches to increasing the value of a food crop are (1) the accumulation of large quantities of a desirable intrinsic nutrient or reduction of undesirable compounds, and (2) accumulation of valuable compounds that are not normally produced in the crop (**Figure 1**).

Agricultural crops with added value, such as those with improved nutritional qualities, are under continuous development. Newell-McGloughlin (2008) reviewed improvements to various crops, including increased protein quantity and quality in maize, potato, rice, and soybean; increased vitamin content (vitamins C, E, or provitamin A) in maize, strawberry, and tomato; increased carotenoid levels (β-carotene, lycopene, or lutein) in rice, potato, and tomato; increased flavonoid levels in maize, rice, tomato, and soybean; increased iron content in rice and lettuce; and decreased glycoside and solanin levels in potato.

Other examples include improvements to the nutritional qualities of rice and tomato. Rice contains little natural β-carotene, a precursor of provitamin A. Golden rice was modified to accumulate β-carotene by the insertion of exogenous genes (Ye et al., 2000). In 2008, two transcription factors from snapdragons were expressed in tomato to enhance anthocyanin accumulation in tomato fruit to levels typically found in blackberries and blueberries (Butelli et al., 2008). These nutritionally and functionally enriched products may be valuable for human and animal health, particularly in situations where availability of nutrients is low. However, where nutritional compounds can be acquired from other foods naturally rich in those nutrients, the market value of nutritionally enhanced foods may not be high enough to justify the economic inputs required. Some economically valuable compounds can be produced that are not commonly found in a daily diet, such as therapeutic components of medicinal plants. The high value of such compounds may allow economically viable production.

Miraculin is a taste-modifying glycoprotein that is extracted from the miracle fruit (*Richadella dulcifica*). It has the unique ability to modify a sour taste into a sweet taste and has potential as a natural, safe, low-calorie alternative to artificial sweeteners for diabetics and people on restricted diets. However, despite its great potential, miracule fruit production is limited because it is a tropical plant that is difficult to cultivate (Kurihara and Nirasawa, 1997). Introduction of the miraculin gene into the tomato genome resulted in the production of tomato fruit that accumulated miraculin (Sun et al., 2007; Hirai et al., 2011a). One gram of miracle fruit pulp typically contains approximately 400 μg of miraculin glycoprotein. Genetically, modified tomatoes accumulated 100–1700 μg of miraculin per gram of fresh weight tomato fruit. Miraculin accumulation varied according to the promoter and terminator used for transgene expression, cultivation conditions, and host cultivar used (Hiwasa-Tanase et al., 2012). Furthermore, miraculin accumulation in modified tomato was more stable in a closed cultivation system than in a netted greenhouse (Hirai et al., 2010). In Japan, a single miracle fruit is worth more than $2. Miraculin-accumulating tomatoes and purified miraculin protein can be produced more cost-effectively than miracle fruit, and the high value of miraculin-producing tomatoes compared to normal tomatoes is sufficient to justify the use of plant factories.

### Agricultural Crops as Edible Medicines

The production of therapeutic recombinant proteins using transgenic plants has been actively promoted due to the numerous potential advantages of this approach (Twyman et al., 2003; Obembe et al., 2011; Ahmad et al., 2012; Xu et al., 2012; Abiri et al., 2015). Compared to mammals and bacteria, plants act as cost-effective bioreactor systems that can be scaled up easily, lack prions, have no human viral pathogen or toxic contamination risks, and have high storage capacities when products accumulate in seeds. Furthermore, purifying and processing costs are limited and labor costs are minimized if the resultant plants can be directly consumed as medicines (Ahmad et al., 2012; Jacob et al., 2013). The commercial production of recombinant proteins using closed cultivation systems may allow more efficient and cost-effective production of feedstocks and bio-pharmaceutical products compared to other production systems. Several recent reviews highlighted the potential of plants for use in molecular farming of desirable compounds such as industrial enzymes and pharmaceutical vaccines and antibodies (Obembe et al., 2011; Ahmad et al., 2012; Xu et al., 2012; Jacob et al., 2013; Abiri et al., 2015; Fahad et al., 2015).

Although the majority of vaccines are administered by injection, vaccines that can be delivered orally and absorbed through the mucosal immune system have practical advantages. Vaccines that are delivered via the mucosal immune system affect both local and systemic immunity, are easy to administer, and are less stressful for patients compared to painful injections. An “edible vaccine” is a crop that accumulates immunogenic antigens in the edible tissue for livestock or human use. There are numerous published studies regarding vaccine production in plants such as potato, lettuce, soybean, maize, tomato, banana, and rice (Obembe et al., 2011; Aswathi et al., 2014; Saxena and Rawat, 2014; Azegami et al., 2015; Fahad et al., 2015). Recently, soybeans that accumulated Alzheimer’s disease vaccine peptides (Maruyama et al., 2014), carrots that produced HIV antigens (Lindh et al., 2014), and rice that accumulated peptide vaccines for pollen allergies (Takaiwa and Yang, 2014) were developed for human use.

In Japan, transgenic strawberries expressing dog interferon-α were commercialized and sold as an oral drug from March 2014. This is the first example of the use of a powdered transgenic plant as a medicine (Tabayashi and Matsumura, 2014). The powdered plant product was effective in the treatment of periodontal disease, and no extraction or purification of the active ingredients was needed. Patent applications were submitted and published in several countries (PTC number PTC/JP2007/050281). The transgenic strawberry is developed from cultivation to drug product in a completely closed cultivation system. Strawberry was selected as the host plant because it can be eaten without being cooked, which is an important advantage for the production of heat-sensitive interferon-α. In addition, strawberry can be readily reproduced by vegetative propagation, which decreases the risk of gene silencing (Stam et al., 1997; Sun et al., 2006; Hirai et al., 2011b), facilitates the development process, and aids production of consistent seed lots. Although, the strawberry plant is considerably less productive with respect to speed of growth and biomass accumulation compared to other plants, the advantages described above made strawberry an ideal subject for development of this medicine.

Overall, molecular farming is an excellent system for utilizing crop characteristics to produce edible medicines with low purification costs (**Figure 1**). Moreover, closed cultivation systems that allow stable and uniform production of a crop are extremely suitable for the consistent production of pharmaceutical and industrial compounds using transgenic plants.

## Crop Traits to Enhance Cultivation in Plant Factories

Features of closed cultivation systems include basal multistage-rack cultivation, soil-free cultivation, optional continuous photoperiod growth, limited light intensity, and low-stress conditions (**Figure 1**). Therefore, many crops developed for cultivation in the field are unsuitable for closed cultivation system. If agricultural crops can be adapted for growth in closed cultivation systems, such as with lettuce, modification of growth patterns to allow high productivity will enhance cost-effectiveness. Furthermore, the specialized cultivars developed for use in closed cultivation systems can be used as host plants for genetic introduction of high-value traits.

There are several crucial factors that must be considered when developing crops for cultivation in enclosed and limited spaces, such as plant size, life-cycle duration, and yield (**Figure 1**). Plants need to be relatively small to suit the closed system, although the importance of this factor depends on the crop. Field rice and wheat cultivars acquired semi-dwarf traits during the “Green Revolution” (Hedden, 2003). Before plants with semi-dwarf traits became available, the stems of tall wheat and rice plants were not strong enough to support the heavy weight of the grain of high-yielding varieties. Therefore, large yield losses occurred as a result of plant lodging. The semi-dwarf trait confers lodging resistance under heavy manuring, and total biomass is increased. High yields can be attained by reducing production loss from lodging. The semi-dwarf genes in rice and wheat were identified from gibberellin (GA)-related mutations. The gene *Reduced height1* (*Rht1*), which is responsible for the semi-dwarf trait in wheat, encodes a negative regulator of the GA response (Peng et al., 1999). The gene *semi-dwarf1* (*sd1*), which is responsible for the semi-dwarf trait in rice, encodes the GA synthesis enzyme GA 20-oxidase (Sasaki et al., 2002; Sakamoto and Matsuoka, 2004). By contrast, the barley dwarfing gene *uzu* is GA-independent. Semi-dwarf barley does not respond to brassinosteroids (BRs), a type of plant steroid hormone. The uzu phenotype is caused by a missense mutation in the BR receptor protein, barley (*Hordeum vulgare*) HvBRI, a homolog of *Arabidopsis* BR-insensitive 1 (BRI1; Chono et al., 2003). The *BRI1* mutant homolog in rice, *OsBRI1*, shows not only the semi-dwarf phenotype but also an erect-leaf phenotype. Similar phenotypes were detected in rice by manipulating the C-22 hydroxylation step in the BR biosynthesis pathway (Sakamoto et al., 2006). The aboveground biomass of BR mutant rice plants was 1.4-fold higher than in WT plants. This increase was attributed to improved photosynthetic efficiency from the increased light penetration to lower leaves afforded by the erect-leaf phenotype (Sakamoto et al., 2006). Manipulation of BR levels and BR sensitivity via modification of other BR-related genes increased yields in rice, barley, cotton, and *Arabidopsis* (Divi and Krishna, 2009; Vriet et al., 2012).

In recent work, the expression of the chimeric repressor for *Arabidopsis* ILI1 binding bHLH (AtIBH1; *P35S:AtIBH1SRDX*), which has the plant-specific transcriptional repression domain SRDX fused to the C-terminus of AtIBH1, induced a dwarf phenotype in *Arabidopsis* and tobacco plants, with reduced cell size (Hiratsu et al., 2003; Ikeda et al., 2012; Nagatoshi et al., 2016). AtIBH1, which is a transcription factor, regulates cell elongation in response to brassinosteroid and gibberellin signaling (Ikeda et al., 2012). The *AtIBH1SRDX* tobacco plants produced four times more biomass per unit of cultivation volume, which means vertical farming that is stacking of multiple shelves for plant growth, compared with wild-type plants (Nagatoshi et al., 2016). When the genes for anti-hepatitis B virus antibodies were expressed in the *AtIBH1SRDX* tobacco plants, the dwarf plants produced about four times more antibody per unit of cultivation volume than in wild-type plants. They showed that *AtIBH1SRDX* is a useful tool for the manipulation of plant phenotype for cost-effective production of high-value products by transgenic plant in closed cultivation systems.

Shortening the life cycles of cultivated plants would increase annual production in a closed system. *Arabidopsis TERMINAL FLOWER 1* (*TFL1*) is a key gene that affects the developmental phases and architecture of *Arabidopsis* (Shannon and Meeks-Wagner, 1991; Alvarez et al., 1992; Ray et al., 1996). The recessive mutant *tfl1* exhibits a terminal flower phenotype and has a significantly shorter vegetative phase than wild-type plants due to its early transition to the reproductive phase (Shannon and Meeks-Wagner, 1991; Bradley et al., 1997). Conversely overexpression of *RCN1* and *RCN2*, rice *TFL1* homologs, revealed delay of phase change from the branch shoot to the floral meristem state (Nakagawa et al., 2002). In tomato, the *SELF-PRUNING* (*SP*) gene, which is a homolog of *TFL1*, regulates switching from vegetative to reproductive phase (Pnueli et al., 1998). The recessive *sp* mutant shows ‘determinate’ trait and the trait confers the short plant height and the short life cycle in comparison with ‘indeterminate’ tomato. Numerous genes that regulate flowering time were previously identified and considered for plant breeding applications (Jung and Müller, 2009; Blümel et al., 2015).

**FIGURE 2:**
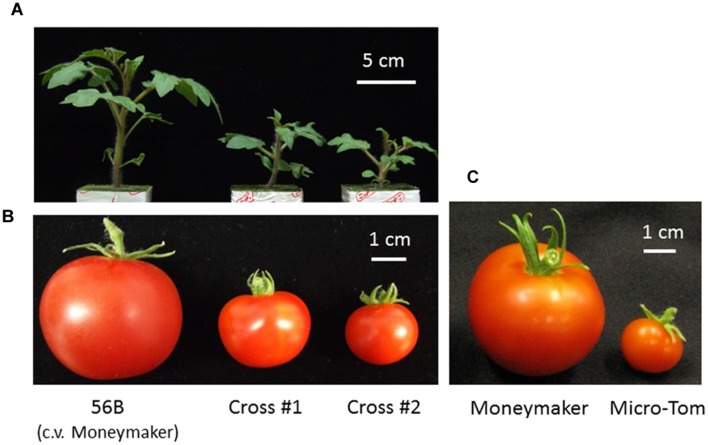
**A tomato developed for cultivation in a closed cultivation system.** The tomato plant 56B (cv. Moneymaker), which accumulates miraculin, has a normal plant size and exhibits an indeterminate type; it was crossed with the dwarf, determinate-type tomato Micro-Tom with the aim of enhancing traits that would be beneficial for growth in a closed cultivation system. Hybrid lines were selected based on plant size, fruit size, determinate inflorescence and miraculin accumu lation, and the lines were named cross #1 and cross #2 (Kato et al., 2010). **(A)** Seedlings at 22 days after germination. **(B,C)** Average size of red fruit. This figure was previously presented in Kato et al. (2010) and has been slightly modified.

The tomato (*Solanum lycopersicum*) cultivar Micro-Tom, which was bred for home gardening purposes, has a miniature growth phenotype. Although Micro-Tom fruits have poor flavor, the cultivar exhibits small features (height approximately 10–20 cm) and can be easily transformed, and it is therefore widely used as a tomato research model (Matsukura et al., 2008; Saito et al., 2011). The dwarf trait in Micro-Tom is attributed to at least two major recessive mutations: *dwarf* (*d*) in the gene encoding the BR biosynthetic enzyme, and *miniature* (*mmt*) in a GA signaling-related gene (Martí et al., 2006; Matsukura et al., 2008). Moreover, the Micro-Tom cultivar has a determinate phenotype derived from a mutation in the *SP*gene. Micro-Tom has a short life cycle (70–90 days from seed germination to fruit maturation) compared with most cultivated tomato varieties (90–110 days) (Saito et al., 2011), exhibits high fertility, and sets a large amount of fruit under normal fluorescent lamps. All of the traits exhibited by Micro-Tom are useful for breeding new plant varieties for use in closed cultivation systems. The genetically modified tomato cultivar “Moneymaker,” which produces and accumulates miraculin as a result of an inserted exogenous gene, is indeterminate and has a normal size. Moneymaker was crossed with Micro-Tom to create a tomato suitable for a closed cultivation system (Kato et al., 2010). Selected hybrids were determinate and smaller than the Moneymaker parent. Hybrid fruits were larger than those of Micro-Tom, and the yield per area was also higher (**Figure 2**).

Luxuriant foliar development and fruit setting under weak light, as well as adaptability to nutriculture systems, contribute to balanced growth in closed environments; however, the factors that determine that these traits are not well-understood. Cultivars or mutants with desirable traits might be identified by screening genetic resources obtained from past breeding programs or from mutagenized populations developed using ethyl methane sulfonate (EMS) or gamma irradiation (TOMATOMA^1^, Genes that Make Tomatoes^2^, Tomato Genetics Resource Center^3^).

## Additional Notes

This review highlights genetic targets that may be of use in molecular breeding programs to develop agricultural crops for profitable cultivation in closed cultivation systems. To create objective traits, a wide variety of genetic information and molecular breeding techniques are used, including marker-assisted selection, quantitative trait locus (QTL) analysis, genetic linkage maps, and transgenic techniques (Jiao et al., 2012; Lusser and Davies, 2013). Whole genomes have been completely sequenced for a variety of cultivated crops, including rice (2005), maize (2009), wheat (2014), soybean (2010), tomato (2012), melon (2012), watermelon (2012), potato (2008), cucumber (2009), and eggplant (2014). These rich sources of sequence information can be readily applied to breeding. Active accumulation of transgenic proteins for pharmaceutical and health purposes can be optimized using several approaches. These include selection of an appropriate host plant, codon optimization, choice of promoter and terminator, use of specific organs and tissues for protein secretion, and expression of the target gene from the nuclear or chloroplast genomes (Twyman et al., 2003; Xu et al., 2012; Abiri et al., 2015). These techniques and tools for molecular breeding make it possible to create agricultural crops with various useful and diverse traits.

In this review, breeding targets were discussed that are likely to be of greatest utility in optimizing plant growth in closed cultivation systems. Several additional traits could also be of use (**Figure 1**). For example, parthenocarpy, which is the production of fruit without fertilization, could reduce workloads and labor costs by reducing inputs needed for consistent pollination. Parthenocarpy is also valuable in the containment of transgenic plant materials. The jointless trait, where no abscission zones are formed on leaves, flowers, or fruit, would reduce product loss through dropping. The free-standing trait, which allows plants to grow without additional support structures, would be conducive to reducing time and material costs. The jointless and free-standing traits may be particularly useful for cultivation systems that employ moving shelves.

## Conclusion

Since the beginning of agriculture, numerous plants have been developed through artificial selection for field cultivation. Modern closed cultivation systems allow environmental conditions to be tightly controlled and ensure the production. The systems offer great potential for production of specialized crops which have additional value. However, crop breeding specifically for closed cultivation systems had been limited to date despite its potential availability. Novel cultivars are required to sympathize with the challenges of agricultural technology. Breeding an indoor suitable cultivar can be accomplished through complex molecular techniques, which property benefits will familiarize closed cultivation systems.

## Author Contributions

All authors listed, have made substantial, direct and intellectual contribution to the work, and approved it for publication.

## Conflict of Interest Statement

The authors declare that the research was conducted in the absence of any commercial or financial relationships that could be construed as a potential conflict of interest.

## Footnotes

1 http://tomatoma.nbrp.jp/

2 http://zamir.sgn.cornell.edu/mutants/

3 http://tgrc.ucdavis.edu/index.aspx
